# Metabolomic profiling of Prader-Willi syndrome compared with essential obesity

**DOI:** 10.3389/fendo.2024.1386265

**Published:** 2024-05-15

**Authors:** Antonello E. Rigamonti, Elisa Polledri, Chiara Favero, Diana Caroli, Adele Bondesan, Graziano Grugni, Stefania Mai, Silvano G. Cella, Silvia Fustinoni, Alessandro Sartorio

**Affiliations:** ^1^ Department of Clinical Sciences and Community Health, University of Milan, Milan, Italy; ^2^ Istituto Auxologico Italiano, Istituto di Ricovero e Cura a Carattere Scientifico (IRCCS), Experimental Laboratory for Auxo-endocrinological Research, Piancavallo-Verbania, Italy; ^3^ Istituto Auxologico Italiano, Istituto di Ricovero e Cura a Carattere Scientifico (IRCCS), Laboratory of Metabolic Research, Piancavallo-Verbania, Italy; ^4^ Fondazione IRCCS Ca’ Granda Ospedale Maggiore Policlinico, Milan, Italy; ^5^ Istituto Auxologico Italiano, Istituto di Ricovero e Cura a Carattere Scientifico (IRCCS), Experimental Laboratory for Auxo-endocrinological Research, Milan, Italy

**Keywords:** Prader-Willi syndrome, essential obesity, metabolomics, biochemical pathways, lipid metabolism

## Abstract

**Introduction:**

Prader-Willi syndrome (PWS) is a rare disease, which shows a peculiar clinical phenotype, including obesity, which is different from essential obesity (EOB). Metabolomics might represent a valuable tool to reveal the biochemical mechanisms/pathways underlying clinical differences between PWS and EOB. The aim of the present (case-control, retrospective) study was to determine the metabolomic profile that characterizes PWS compared to EOB.

**Methods:**

A validated liquid chromatography-tandem mass spectrometry (LC-MS/MS) targeted metabolomic approach was used to measure a total of 188 endogenous metabolites in plasma samples of 32 patients with PWS (F/M = 23/9; age: 31.6 ± 9.2 years; body mass index [BMI]: 42.1 ± 7.0 kg/m^2^), compared to a sex-, age- and BMI-matched group of patients with EOB (F/M = 23/9; age: 31.4 ± 6.9 years; BMI: 43.5 ± 3.5 kg/m^2^).

**Results:**

Body composition in PWS was different when compared to EOB, with increased fat mass and decreased fat-free mass. Glycemia and HDL cholesterol were higher in patients with PWS than in those with EOB, while insulinemia was lower, as well as heart rate. Resting energy expenditure was lower in the group with PWS than in the one with EOB, a difference that was missed after fat-free mass correction. Carrying out a series of Tobit multivariable linear regressions, adjusted for sex, diastolic blood pressure, and C reactive protein, a total of 28 metabolites was found to be associated with PWS (vs. non-PWS, i.e., EOB), including 9 phosphatidylcholines (PCs) ae, 5 PCs aa, all PCs aa, 7 lysoPCs a, all lysoPCs, 4 acetylcarnitines, and 1 sphingomyelin, all of which were higher in PWS than EOB.

**Conclusions:**

PWS exhibits a specific metabolomic profile when compared to EOB, suggesting a different regulation of some biochemical pathways, fundamentally related to lipid metabolism.

## 1 Introduction

Prader-Willi syndrome (PWS) is a rare disease, which shows a variable clinical phenotype, including muscle hypotonia and failure to thrive during infancy, short stature, intellectual disability, behavioral abnormalities, hyperphagia, and obesity in childhood and adulthood (1, 2). Apart from growth hormone (GH) deficiency (GHD) (3), other endocrine diseases are frequently observed, including hypothyroidism and hypogonadism (4).

PWS is due to some abnormality in the so-called Prader-Willi critical region (PWCR) at 15q11-q13. In particular, three main genetic patterns have been identified: paternal 15q11-q13 deletion (del15) (65-75% of cases), maternal uniparental disomy 15 (UPD15) (20-30% of cases), and imprinting center defect (1-3%) (5).

Though clinical studies with conflicting results have been published, PWS-associated obesity exhibits clinical characteristics that are somewhat different from those of essential obesity (EOB), including body composition (particularly, adipose tissue), neuroendocrine system (e.g., ghrelin, oxytocin, and adiponectin), response to body weight reduction programs, occurrence of obesity-related comorbidities such as type 2 diabetes mellitus (T2DM), and long-term cardiovascular prognosis (6–12).

Evaluation of metabolome, i.e., the total of metabolites that, in the human organism, serve as substrates or by-products of enzymatic or non-enzymatic reactions, has become, in the last decade, a valuable tool to understand the biochemical pathways that are altered in patients affected by specific diseases. With the spread and technological improvements of -omics platforms, it is now possible to characterize the metabolomic profiling in manifold biofluids, including plasma and urine samples, which can be easily and repeatedly collected in any subject. This methodological approach is extremely attractive due to the potential diagnostic and/or therapeutic implications (13).

Surprisingly, while a huge number of metabolomics-based clinical studies have been carried out in EOB (14), to the best of our knowledge, PWS has never been investigated by this innovative methodology so far.

So, the aim of the present (case-control, retrospective) study was to compare the metabolomic profiles in patients with PWS vs. EOB. We hypothesized that a different metabolome, compared to EOB, could explain some aspects of the biochemistry and, so, the peculiar clinical phenotype in PWS.

## 2 Materials and methods

### 2.1 Study design

The present case-control study was retrospective, based on the use of plasma samples already available.

Before enrolment, patient selection was the initial phase of the study to evaluate inclusion/exclusion criteria and to verify the presence of the patient’s consent (see below for details).

### 2.2 Subjects

Adults of both sexes were selected from the patients’ population admitted to the Division of Auxology (PWS) and the Division of Metabolic Diseases (EOB) of Istituto Auxologico Italiano, Piancavallo-Verbania, Italy, for a 3-week in-hospital multidisciplinary body weight reduction program.

First of all, patients affected by PWS were selected; then, age-, sex- and body mass index (BMI)-matched patients affected by EOB were identified and included in the control group.

PWS patients showed the typical clinical phenotype of the syndrome, this diagnosis being confirmed by cytogenetic analysis (del15 = 20 cases; UPD15 = 12 cases).

The inclusion criteria were: (1) individuals of both sexes, aged ≥18 years; (2) individuals with or without metabolic syndrome (see below for its definition); (3) individuals having a BMI >30 kg/m^2^ (for both groups, i.e., PWS and EOB). The exclusion criteria were: (1) secondary causes of obesity (e.g., steroid-induced obesity), apart from PWS; (2) individuals with systolic blood pressure (SBP) ≥180 mmHg and diastolic blood pressure (DBP) ≥110 mmHg; (3) cardiovascular, psychiatric, neurological, or other (relevant) medical diseases evident in the previous 6 months; (4) individuals (and/or their parents) who refused to sign the consent form.

The study protocol was approved by the Ethical Committee (EC) of the Istituto Auxologico Italiano, IRCCS, Milan, Italy (EC code: 2023_03_21_08; research project code: 01C316; acronym: METABOPWS).

### 2.3 Resting energy expenditure

Resting energy expenditure (REE) was determined after an overnight fast using an open-circuit, indirect computerized calorimetry (Vmax 29, Sensor Medics, Yorba Linda, Ca, USA) with a rigid, transparent, ventilated canopy.

### 2.4 Anthropometric measurements

A scale with a stadiometer was used to determine height (with a precision of 0.1 cm) and weight (with a precision of 0.1 kg) (Wunder Sa.Bi., WU150, Trezzo sull’Adda, Italy). Waist circumference (WC) was measured with a flexible tape in a standing position, halfway between the inferior margin of the ribs and the superior border of the crista, while hip circumference (HC) was measured at the largest parts around the buttocks. Body composition was measured by bioimpedance analysis (Human-IM Scan, DS-Medigroup, Milan, Italy) after 20 minutes of supine resting. BMI (weight in kg divided by height in meters squared), fat mass (FM), and fat-free mass (FFM) were determined in all subjects.

### 2.5 Biological sample collection

Blood samples were collected from patients, following a standardized protocol, at the beginning of the body weight reduction program (T0). The same types of tubes and consumables for each cluster of parameters were used throughout the entire duration of the study to improve consistency.

Blood samples were collected in lithium heparin tubes at around 8:00 AM after an overnight fast. Cells were separated from plasma with centrifugation (20-24°C for 10 minutes at 2500 g) within 2 hours from the blood collection. Plasma was then transferred in pre-cold tubes and put in ice to preserve the metabolome. Each tube was vortexed for at least 10 seconds, divided into aliquots, and stored at -20°C.

Plasma samples were delivered from our Laboratories, located in Piancavallo-Verbania, to Milan while keeping the samples frosted, where they were stored at -20°C until the metabolomic analyses.

### 2.6 Metabolic, biochemical

Total cholesterol (T-C), high-density lipoprotein cholesterol (HDL-C), low-density lipoprotein cholesterol (LDL-C), triglycerides (TG), glucose, insulin, and C-reactive protein (CRP) were measured.

Colorimetric enzymatic assays (Roche Diagnostics, Monza, Italy) were used to determine serum T-C, LDL-C, HDL-C, and TG levels. The sensitivities of the assays were 3.86 mg/dL [1 mg/dL = 0.03 mmol/L], 3.87 mg/dL [1 mg/dL = 0.03 mmol/L], 3.09 mg/dL [1 mg/dL = 0.03 mmol/L] and 8.85 mg/dL [1 mg/dL = 0.01 mmol/L], respectively.

Serum glucose level was measured by the glucose oxidase enzymatic method (Roche Diagnostics, Monza, Italy). The sensitivity of the method was 2 mg/dL [1 mg/dL = 0.06 mmol/L]. Serum insulin concentration was determined by a chemiluminescent immunometric assay, using a commercial kit (Elecsys Insulin, Roche Diagnostics, Monza, Italy). The sensitivity of the method was 0.2 µU/mL [1 µU/mL = 7.18 pmol/L].

The intra- and inter-assay coefficients of variation (CVs) were the following: 1.1% and 1.6% for T-C, 1.2% and 2.5% for LDL-C, 1.8% and 2.2% for HDL-C, 1.1% and 2.0% for TG, 1.0% and 1.3% for glucose, and 1.5% and 4.9% for insulin.

CRP was measured using an immunoturbidimetric assay (CRP RX, Roche Diagnostics GmbH, Mannheim, Germany). The sensitivity of the method was 0.03 mg/dL.

For each patient, the homeostatic model assessment of insulin resistance (HOMA-IR) was also calculated, according to the following formula: (insulin [μU/mL] × glucose [mmol/L])/22.5 (15).

### 2.7 Evaluation of blood pressure

Blood pressure was measured on the right arm, using a sphygmomanometer with appropriate cuff size, with the subject in a seated position and relaxed condition. The procedure was repeated three times at 10 min intervals in between; the means of the three values for SBP and DBP were recorded.

### 2.8 Definition of metabolic syndrome

According to the International Diabetes Federation (IDF) criteria for diagnosis of metabolic syndrome in adults (16), our patients were considered positive for the presence of metabolic syndrome if they had abdominal obesity plus two or more of the following factors: (i) increased TG level, (ii) reduced HDL-C levels, (iii) increased BP levels, (iv) increased fasting glucose levels or previously diagnosed T2DM. For cut-offs of each parameter and further information (e.g., use of lipid/glucose-lowering drugs) see ref (16).

### 2.9 Metabolomics analyses

The metabolomics profile of plasma samples collected from subjects was assessed with a targeted approach, in particular a liquid chromatography-tandem mass spectrometry method (LC–MS/MS, Sciex 550 Qtrap) implementing the Absolute IDQ p180 kit (Biocrates Life Sciences AG, Innsbruck, Austria). With this method, a total of 188 metabolites was quantified, among which 21 amino acids, 21 biogenic amines, the sum of hexose (H1), 40 acylcarnitines, 15 sphingolipids, and 90 glycerophospholipids among which 14 lyso-phosphatidylcholines (LysoPC), 38 diacylphosphatidylcholine (PC aa), and 38 acyl-alkylphosphatidylcholine (PC ae). The analytical details used in our analyses were extensively reported previously (17).

### 2.10 Data elaboration and statistical analyses

Demographic, biochemical, and clinical parameters were expressed as frequency and percentage for categorical variables and in terms of mean ± standard deviation (SD) or median and first quartile — third quartile [q1;q3] as appropriate for continuous variables. The normal distribution of continuous variables was tested by graphical inspection.

The differences in each parameter between patients with PWS and those with EOB were analyzed by the t-test or Wilcoxon sum-rank test for continuous variables. In the case of a categorical variable, the chi-squared test was applied.

For each metabolite, data <LOD were replaced with the minimum limit of detections (LOD) values divided by 2 (i.e., LOD/2); then, descriptive statistics were performed, including mean ± SD and the following percentiles: 5°, 25°, 50°, 75° and 95°. Only metabolites with at least 20% of observations greater than the LOD were considered for the following statistical analyses. Metabolite concentrations were log-transformed (base e) to ensure normal distribution and then standardized by performing an auto-scale (i.e., each value was subtracted by the mean and divided by the SD), to make the fold-change (FC) of all different metabolites comparable.

We used Tobit regression models to evaluate the differences in the metabolite concentrations between PWS and EOB. This approach was chosen because the Tobit regression model is designed to estimate linear relationships with left- or right-censoring dependent variables (18, 19). In our case, metabolite concentrations lower than the LOD were considered as the left-censored values. A Tobit regression model was applied for each metabolite: the standardized natural logarithm of the metabolite concentrations was the dependent variable, while the independent variable of interest was the condition of PWS (or non-condition of PWS, i.e., EOB). In the multivariable models, gender, DBP, and CRP were chosen as adjustment covariates, considering the results of previous univariate Tobit linear regressions, built to evaluate the relationships of each metabolite concentration with demographic, biochemical, and clinical characteristics in the entire population. From each multivariable Tobit model, we estimated the geometric means for the PWS group and the control groups (EOB) with the relative confidence interval (95% CI). The ratio of the geometric means was used to obtain the Fold-Change (FC). Due to the high number of comparisons, we applied a multiple comparison correction method based on the Benjamini–Hochberg False Discovery Rate (FDR) to calculate the FDR-adjusted p-value (FDR p-value).

A volcano plot of log_2_ (FC) vs -log_10_(FDR p-value) was used to display the results. Only for the 28 analytes with significant FDR p-values we reported the box-plots to visualize the original distribution of metabolite concentrations (without standardization) in PWS and EOB groups.

Analyses were performed using SAS version 9.4 (SAS Institute, Cary, NC).

## 3 Results

Patients with PWS (F/M = 23/9; age: 31.6 ± 9.2 years; BMI: 42.1 ± 7.0 kg/m^2^) and with EOB (F/M = 23/9; age: 31.4 ± 6.9 years; BMI: 43.5 ± 3.5 kg/m^2^) were recruited, being the two groups comparable in terms of sex, age, BMI, and metabolic syndrome prevalence. **Table 1** reports descriptive statistics of demographic, biochemical, and clinical data referred to patients with PWS or EOB, including the corresponding inter-group comparisons.

**Table 1:** Demographic, biochemical, and clinical characteristics of patients with PWS and EOB.

Characteristic		PWS	EOB	P-value
		N=32 - CASES	N=32 - CONTROLS	
Age (years)		31.6 ± 9.2	31.4 ± 6.9	0,9003
Sex	M	9 (28.1%)	9 (28.1%)	1
	F	23 (71.9%)	23 (71.9%)
BMI (kg/m^2^)		42.1 ± 7.0	43.5 ± 3.5	0,2963
Waist circumference (cm)		122.6 ± 14.5	117.5 ± 10.6	0,1101
FFM (kg)		45.6 ± 7.1	57.9 ± 14.4	<0.0001
FFM (%)		48.9 ± 8.1	48.3 ± 5.3	0,7576
FM (kg)		51.5 ± 13.4	61.4 ± 14.4	0,0060
FM (%)		52.4 ± 6.0	51.8 ± 5.5	0,7028
mREE (kcal)		1499 ± 359	2133 ± 357	<0.0001
mREE/FFM (kcal/kg)		33.4 ± 7.8	36.0 ± 4.1	0,0991
SBP (mmHg)		126 ± 12.5	128 ± 10.1	0,4431
DBP (mmHg)		80 ± 3.7	82.5 ± 8.9	0,1759
Antihypertensive drugs	Yes	5 (15.6%)	5 (15.6%)	1
	No	27 (84.4%)	27 (84.4%)
HR (bpm)		71.5 ± 11.8	86.2 ± 9.9	<0.0001
Glucose (mg/dL)		103 ± 29	87 ± 9.6	0,0052
Insulin (mU/L)		14.8 ± 6.6	21.8 ± 10.1	0,0019
HOMA-IR		4.7 ± 2.2	3.8 ± 2.1	0,1163
HbA1c (%)		7.9 ± 4.2	5.3 ± 0.3	0,1685
Hypoglycemic drugs	Yes	11 (34.4%)	4 (12.5%)	0,0389
	No	21 (65.6%)	28 (87.5%)
Total cholesterol (mg/dL)		180.4 ± 30.8	164.7 ± 32.4	0,0501
HDL cholesterol (mg/dL)		48.4 ± 11.4	41.7 ± 10.9	0,0186
LDL cholesterol (mg/dL)		117.1 ± 26.1	104.8 ± 30.6	0,0900
Lipid-lowering drugs	Yes	7 (21.9%)	5 (15.6%)	0,5218
	No	25 (78.1%)	27 (84.4%)
TG (mg/dL)		99 [85.5;156.5]	119 [90;144]	0,6969
CRP (mg/dL)		1.1 [0.5;1.7]	0.5 [0.4;1.2]	0,0713
AST (U/L)		16 [15;21]	19 [16;26]	0,1232
ALT (U/L)		19 [14.5;24]	25 [16;37]	0,1082
Gamma GT (U/L)		18 [12.5;26.5]	21.5 [15;31.5]	0,2532
GH therapy	Yes	20 (62.5%)	1 (3.1%)	<0.0001
	No	12 (37.5%)	31 (96.9%)

Continuous variables were expressed as mean ± SD (p-value from t-test) or as median [q1;q3] (p-value from Wilcoxon rank-sum test) as appropriate; discrete variables were reported as frequency and percentage (p-value from chi-squared test).

For abbreviations see the list included in the text.

When considering body composition, patients with PWS exhibited lower FFM than those with EOB (p<0.0001), as well as a lower FM (p=0.0060), being similar the corresponding values expressed in percentage. REE was lower in patients with PWS than in those with EOB (p<0.0001), a difference that was missed after correcting the REE value by the subject’s FFM (i.e., energy efficiency).

Though SBP and DBP were similar in both groups (PWS and EOB), without any difference in the percentages of subjects treated with antihypertensives, HR was lower in patients with PWS than in those with EOB (p<0.0001).

When considering glucometabolic homeostasis, glycemia and insulinemia were, respectively, higher and lower in patients with PWS than in those with EOB (glucose: p=0.0052; insulin: =0.0019), though the percentage of the subjects treated with antidiabetic drugs was higher in the former than the latter ones (p=0.0389). Nevertheless, there were no differences in HOMA-IR and HbA1c when comparing the two groups (i.e., PWS vs. EOB).

While there were no differences in T-C, LDL-C, and TG, HDL-C levels were higher in patients with PWS than in those with EOB (p=0,0186), being the two groups similarly treated with antilipidemic drugs.

**Supplementary Table S1**, included in the **Supplementary Material**, reports descriptive statistics of all metabolites measured in the plasma for the two groups.

Carrying out Tobit *univariate* linear regression models, several metabolites belonging to different chemical classes were identified to be associated with PWS, including glycerophospholipids (7 lysoPCs a, 12 PCs aa, 13 PCs ae, all lysoPCs and all PCs), sphingolipids (1 sphingomyelin, SM) and 3 acylcarnitines, all of which were higher in patients with PWS than in those with EOB (at least p<0.05) (**Supplementary Table S2** in the **Supplementary Material**).

Carrying out Tobit *multivariable* linear regression models, adjusted for sex, DBP, and CRP, several metabolites belonging to different chemical classes were identified to be associated with PWS, including 7 lysoPCs a, 9 PCs ae, 5 PCs aa, all lysoPCs, all PCs aa, 4 acylcarnitines, and 1 SM, all of which were higher in PWS than EOB (at least p<0.05) (**Table 2** for statistically significant metabolites and **Supplementary Table S3** for statistically non-significant metabolites).

**Table 2:** Multivariable Tobit regression models used to evaluate the association of PWS with metabolites expressed in at least 20% of the population.

Metabolite	N obs < LB	PWS (N=32)			EOB (N=32)			FC	P-value	FDR P-value
		Geometric Mean	95% CI		Geometric Mean	95% CI				
C10	9	1,983	1,371	2,869	0,579	0,402	0,832	3,43	<0.0001	0,0001
lysoPC a C20:3	0	1,722	1,243	2,386	0,608	0,444	0,831	2,83	<0.0001	0,0001
C14:1	20	1,808	1,141	2,866	0,405	0,249	0,658	4,47	<0.0001	0,0002
lysoPC a C17:0	9	1,945	1,326	2,854	0,604	0,415	0,879	3,22	<0.0001	0,0002
C5	17	1,563	1,010	2,419	0,407	0,260	0,639	3,84	<0.0001	0,0003
lysoPC a C18:2	0	1,512	1,073	2,132	0,607	0,437	0,845	2,49	0,0001	0,0016
lysoPC a C16:1	0	1,532	1,080	2,173	0,613	0,438	0,857	2,50	0,0001	0,0017
lysoPC_s	0	1,544	1,082	2,203	0,670	0,476	0,942	2,31	0,0004	0,0079
PC ae C36:4	0	1,582	1,143	2,190	0,756	0,553	1,032	2,09	0,0006	0,0108
lysoPC a C16:0	0	1,564	1,092	2,239	0,703	0,498	0,993	2,22	0,0008	0,0127
PC ae C38:5	0	1,615	1,151	2,267	0,766	0,553	1,061	2,11	0,0009	0,0133
PC ae C32:1	0	1,499	1,081	2,079	0,739	0,540	1,011	2,03	0,0011	0,0145
lysoPC a C18:1	0	1,385	0,969	1,980	0,642	0,456	0,905	2,16	0,0011	0,0145
PC aa C34:1	0	1,550	1,125	2,135	0,786	0,578	1,070	1,97	0,0014	0,0163
PC aa C36:3	0	1,530	1,105	2,119	0,776	0,568	1,061	1,97	0,0016	0,0177
PC ae C36:3	0	1,566	1,121	2,189	0,794	0,576	1,095	1,97	0,0021	0,0216
PC aa C36:2	0	1,647	1,163	2,332	0,815	0,584	1,139	2,02	0,0022	0,0216
lysoPC a C26:0	27	1,163	0,631	2,144	0,320	0,164	0,623	3,64	0,0023	0,0216
PC aa_s	0	1,523	1,099	2,112	0,797	0,582	1,090	1,91	0,0027	0,0236
SM (OH) C22:2	23	1,401	0,799	2,458	0,463	0,259	0,830	3,02	0,0044	0,0329
PC ae C36:5	0	1,501	1,067	2,111	0,789	0,569	1,095	1,90	0,0044	0,0329
PC ae C42:0	2	1,287	0,906	1,830	0,666	0,474	0,934	1,93	0,0046	0,0329
PC ae C34:1	0	1,488	1,075	2,062	0,811	0,593	1,110	1,83	0,0049	0,0329
C3-DC (C4-OH)	30	1,112	0,576	2,148	0,292	0,137	0,622	3,81	0,0049	0,0329
PC ae C36:0	0	1,442	1,013	2,054	0,748	0,533	1,050	1,93	0,0049	0,0329
PC aa C32:0	0	1,484	1,082	2,037	0,834	0,615	1,130	1,78	0,0058	0,0372
PC ae C34:2	0	1,503	1,074	2,103	0,821	0,595	1,134	1,83	0,0065	0,0400
PC aa C38:5	0	1,528	1,077	2,167	0,821	0,587	1,148	1,86	0,0072	0,0426

Sex, CRP, and DBP were selected as adjustment covariates. The statistically non-significant metabolites are reported in the **Supplementary Table S3**. See the **Supplementary Material**. LB=Lower Bound; FC, Fold-Change.

**Figure 1:**
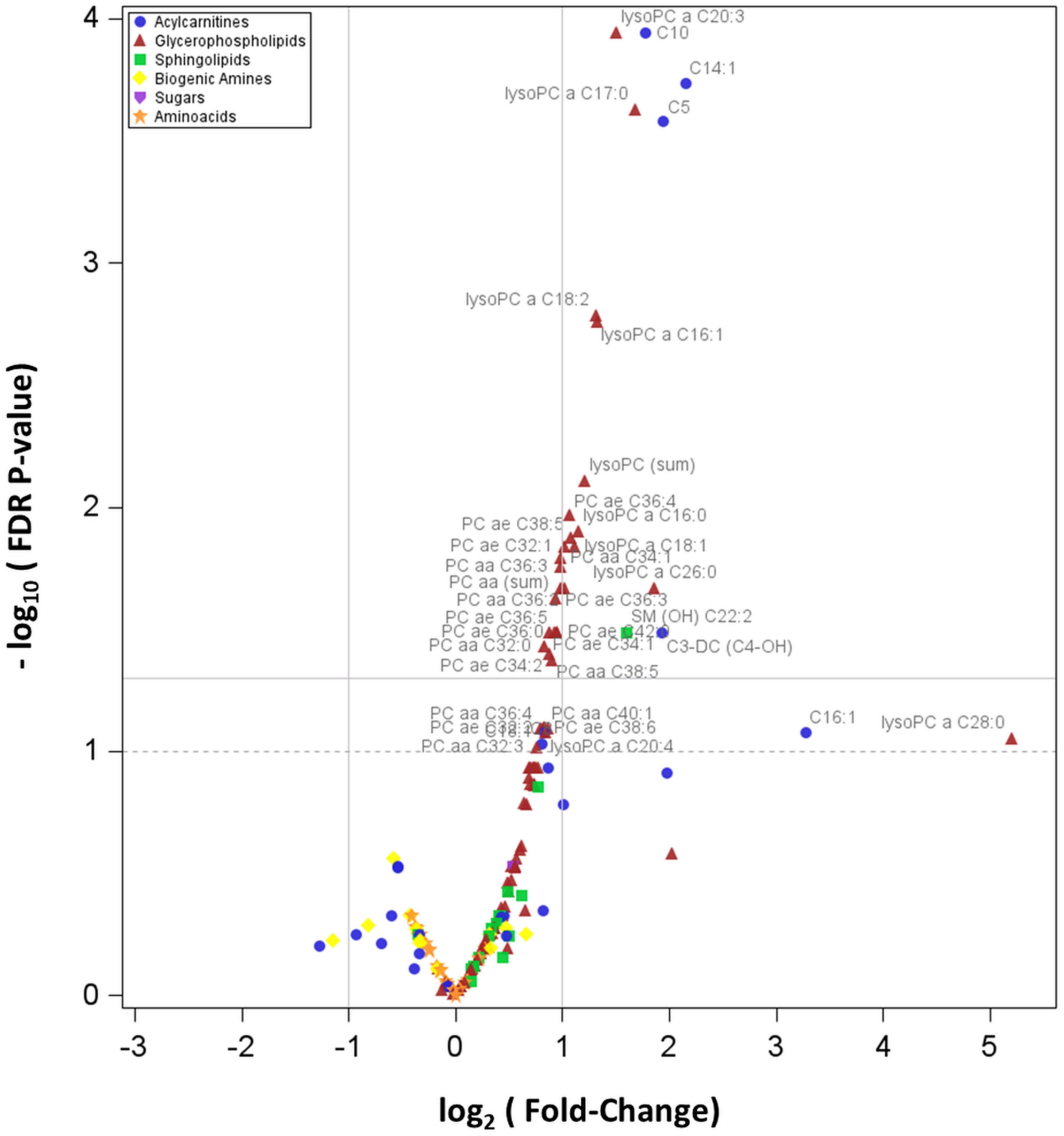
Volcano plot showing how plasma concentrations of each metabolite are differentially expressed between PWS and EOB groups. In particular, the graph reports the values of the 166 detectable metabolites. The horizontal line states the –log_10_ of the FDR 0.05, being the horizontal dot line the –log_10_ of the FDR p-value 0.10; the two vertical lines indicate the log_2_ of the Fold-Change (FC) values 0.5 and 2.0.

**Figure 1** shows the Volcano plot of metabolomics data, while **Figure S1**, included in the **Supplementary Material**, represents the box-plots referring to the distribution of the 28 metabolites associated with PWS (as described above).

## 4 Discussion

In the present study, the plasma metabolomic profile of adult patients with PWS has been determined in comparison with that of a control group, consisting of sex-, age- and BMI-matched subjects with EOB, being similar the prevalence of metabolic syndrome in both groups. When considering the results of a series of multivariable linear regressions, adjusted for some covariates such as sex, DBP, and CRP, plasma levels of many lysoPCs and PCs, together with those of some acylcarnitines and one SM, were shown to be associated with PWS (vs. non-PWS, i.e., EOB), with a FC ranging from 1.83 to 4.47, indicating higher levels of all metabolites in PWS than EOB group.

To the best of our knowledge, no one has so far applied a metabolomic methodology to (biochemically) investigate the peculiar clinical phenotype in PWS compared to EOB. For the first time, in the limitations of our small patients’ sample, we have fundamentally documented an alteration in lipid metabolism, particularly the biochemical pathways of acylcarnitines and lysoPCs/PCs.

Understanding the molecular mechanism(s) underlying metabolomics-derived biochemical results is not easy, but, in the present discussion, we would like to tentatively provide some explanations.

As well-known, obesity is characterized by a lipid excess, with increased plasma non-esterified fatty acids (NEFAs), with ensuing lipid accumulation into different peripheral tissues, such as the liver and skeletal muscle (20, 21). This deleterious process occurs not only as a result of increased NEFA availability, but also reduced FA oxidation (FAO) (22). Indeed, the rate of FAO, the so-called mitochondrial β-oxidation, has been reported to be reduced in the obese state, particularly during fasting (23). The proposed mechanism underlying this reduction is malonyl-CoA inhibition of carnitine-palmitoyl-transferase-1 (CPT1)-mediated entry of NEFA into mitochondria through acylcarnitine translocase (24). This molecular explanation is apparently congruent with our finding of increased C3-DC-acyl carnitine (i.e., malonylcarnitine), which would suggest an intracellular availability of malonyl-CoA.

Anyway, as demonstrated by animal and human studies (25), in obesity there is an “intrinsic” decrease in mitochondrial FAO capacity rather than an acylcarnitines generation (26).

In the present study, a general increase in plasma acylcarnitines levels was found in both EOB and PWS groups (when compared to the corresponding ranges in the normal-weight population) (17, 27), suggesting an impairment in mitochondrial FAO capacity rather than CPT1 function. Furthermore, malonyl-CoA, an inhibitor of CPT1, has not the same biochemical role as malonylcarnitine (C3-DC-acylcarnitine), generated by CPT1.

The acylcarnitine profile, as determined through our metabolomic approach, includes substrates of both fatty acid oxidation and amino acid β-oxidation (28). In particular, long-chain acylcarnitines, containing up to 20 carbons, accumulate in response to incomplete/inefficient FAO. In contrast, amino acid catabolism produces short-chain acylcarnitine species such as C3-acylcarnitine, C4-acylcarnitine, and C5-acylcarnitine. Importantly, the accumulation of medium- and short-chain acylcarnitines can also derive from the intermediate and ultimate steps (or rounds) of an incomplete/inefficient FAO (29). In the present study, differently from patients with EOB, increased levels of medium- (i.e., C10 and C14:1) and short-chain (i.e., C3-DC, C4-OH, and C5) acylcarnitines were found in the group of patients with PWS, suggesting a more incomplete/inefficient mitochondrial FAO, even in the intermediate/ultimate steps of the oxidative shortening of the acyl chain.

To date, we are unable to explain the molecular reasons for this different impairment of FAO in EOB (initial steps of FAO) vs. PWS (intermediate/ultimate steps of FAO). In this regard, our patients with PWS had less FFM (kg) in comparison to patients with EOB (30, 31). If FAO is predominantly carried out by skeletal muscle (32–34), the increased plasma short-chain acylcarnitines levels, found in our subjects with PWS, might depend on an incomplete/inefficient FAO related to a reduced amount of skeletal muscle (22).

Reportedly, plasma levels of branched-chain amino acid (BCAA) and short-chain acylcarnitines are increased in the obese state, mainly due to a dysfunctional BCAA catabolic pathway (27, 35). In particular, C5-acyl-CoA (i.e., isovaleryl-CoA) derives from leucine and isoleucine catabolism, from which C5-acylcarnitine (i.e., isovalerylcarnitine) is generated. In patients with PWS, a more evident sarcopenia has been documented due to GHD, hypogonadism, and muscle hypotonia (36), suggesting a skeletal muscle catabolic or anti-anabolic state that might represent another explanation for the higher short-chain acylcarnitines levels in patients with PWS than in those with EOB.

Lecithin cholesterol acyl transferase (LCAT) is a plasma enzyme that esterifies cholesterol. This enzyme, primarily produced in the liver, is associated with specific lipoproteins, being the majority bound to HDL-C and, to a lesser extent, to LDL-C (37). LCAT has two different catalytic activities that account for its ability to esterify cholesterol: the first is a phospholipase A_2_ activity, which cleaves FAs from the s*n*-2 position of PCs; the second is a transesterification activity, which transfers the cleaved FA to the hydroxyl group on the A-ring of cholesterol, with the production of a molecule of lysoPC. Apolipoprotein A-I (apoA-I), which is predominantly expressed in HDL-C, is an activating factor of LCAT by modifying the presentation of its lipid substrates. As cholesteryl esters are more hydrophobic than free cholesterol, cholesteryl esters formed by LCAT transfer from the surface of lipoproteins to the hydrophobic core. This process transforms the small pre-β-HDL (the so-called nascent HDL) into mature HDL (i.e., larger, spherical-shaped α-migrating particles) (38).

Based on the metabolomic results obtained in the present study, the higher levels of lysoPCs and PCs are supposed to be related to an increase in (HDL-associated) LCAT activity in PWS than EOB. This putative explanation is congruent with the higher HDL-C levels in patients with PWS than in those with EOB.

After its esterification by LCAT, cholesteryl esters on HDL can be transferred to apoB-containing lipoproteins (such as LDL) by cholesteryl ester transfer protein (CETP) (38). In the present study, though the statistical significance was not reached, LDL-C and T-C levels were higher in patients with PWS than in those with EOB. This finding might be an indirect consequence of LCAT hyperactivity, cholesteryl esters enriched HDL and enhanced CETP-mediated transfer of cholesteryl esters from HDL to LDL, with the ensuing increase in T-C levels.

More difficult is to identify the causes of this (supposed) PWS-related LCAT hyperactivity (vs. EOB). These might be found among several factors recognized as specific for PWS compared to EOB, including abundance, distribution, and type of FM, the elevation of adiponectin and ghrelin and reduction of oxytocin and irisin levels, GHD, and impairment in the autonomic nervous system, all of which have been demonstrated to exert some effects on lipid metabolism (such as lysoPcs/PCs, NEFA, HDL-C, *etc*) (6–12). Based on the available information about the PWS patients recruited in the present study, apart from GHD and GH therapy, we cannot determine which factor(s) is of utmost importance, and what is the underlying mechanism(s) of PWS-specific metabolomic differences (vs. EOB) as above described.

As known, SMs represent the major sphingolipid species in mammalian cells, concentrated in the outer leaflet of the plasma membrane (39). SM plays an important role in maintaining the function and integrity of lipid rafts, which are microdomains in the plasma membrane that are implicated in signal transduction (40, 41). SM is synthesized from ceramide (Cer) and PC by sphingomyelin synthases (SMSs), for which two genes have been identified (i.e., SMS_1_ and SMS_2_). Mammalian cells produce many species of SM (42, 43). Liver SMS_2_ is one of the determinants of plasma SM levels (44). Finally, high plasma SM levels have been associated with coronary artery disease, atherosclerosis, and obesity (45–49).

Among several sphingoid metabolites that were measured in the plasma through our metabolomic approach, only one SM, precisely SM C22:2, was found to be associated with PWS (compared to EOB). We do not know why SM C22;2 levels were higher in patients with PWS than in those with EOB. The most plausible explanation is the general abundance of PCs in PWS compared to EOB, which, as described above, represent substrates for SMS_2_.

Another hypothesis can be argued: as sphingolipids are known to elicit cellular inflammatory and specifically immune responses *via* many different molecular mechanisms (46, 50), the distinctive alteration of SM metabolism might be the consequence (or the cause?) of that anomalous activation of the innate immune system that, in PWS, has been demonstrated to be independent from central adiposity and insulin resistance (51).

In recent years, several studies have documented that PWS patients have lower insulinemia and insulin resistance than (BMI-matched) EOB controls, suggesting protected glucose metabolism (11). According to this view, the prevalence of impaired glucose intolerance, T2DM, or metabolic syndrome in PWS tends to be lower when compared to that in EOB controls (6, 10, 52).

In the present study, PWS patients, though more treated with antidiabetic drugs, exhibited lower insulinemia in the context of hyperglycemia, when compared to the group with EOB. No other differences in T2DM biochemical markers, such as HOMA-IR and HbA1c, were found between the PWS and EOB groups. Our results seem to conflict with the commonly accepted view of a favorable glucometabolic homeostasis in PWS (vs. EOB) (11), but, due to the experimental design adopted for the present study, we were forced to strictly match the most relevant demographic, biochemical, and clinical characteristics between PWS and EOB groups, including the diagnosis of metabolic syndrome. This might have introduced an intrinsic (non-avoidable) bias. Furthermore, if abundance, distribution, and type of FM differ in PWS compared to EOB, BMI-matching might have minimized, as demonstrated by the present study and other ones, the typical insulin sensitivity in PWS. Being BMI (as well as FM/FFM) a “gross” parameter, only the use of sophisticated methods capable of evaluating the specific body fat patterning in PWS might solve this issue.

In a recent study by Hou et al. (53), a serum lipidomics analysis was simultaneously explored in PWS, EOB, and normal-weight Chinese children. Results indicated that the total PCs and lysoPCs were deceased in PWS children compared with both EOB and normal-weight groups. In contrast, several SM, Cer, acylcarnitine, and TG species were increased. We do not know the reason(s) for the discrepancy between these results and those reported in our study. Age (children vs. adults), BMI (with higher values in our study), ethnicity (Asian vs. Caucasian), and selection of different gut microbial populations for diet and other environmental factors might be invoked.

Before closing, apart from the limited sample size and the cross-sectional design, some other limitations of our study should be mentioned. First of all, we have determined only the metabolomic profiling in fasting conditions with no specific external interventions. In particular, metabolomic responses to exercise or weight loss might be different in PWS compared to EOB; thus, future studies are mandatory to investigate these research topics. Secondly, we have not determined microbial profiling in feces from any recruited subject. So, we cannot rule out that the changes in metabolite concentrations that were observed in the present study may be related to a different gut microbiota, capable of modifying the host’s metabolism or metabolite absorption from the gut in PWS compared to EOB.

## 5 Conclusions

By using a metabolomic approach, patients with PWS, compared to those with EOB, exhibit higher plasma levels of specific metabolites belonging to some chemical classes, including manyfold lysoPCs/PCs, few (particularly, short-chain) acylcarnitines, and one SM, suggesting a different regulation of some biochemical pathways, fundamentally related to lipid metabolism. Deciphering the molecular mechanisms underlying the specific metabolomic profile might allow us to understand the peculiar clinical phenotype in patients with PWS (vs. those with EOB). Further studies are mandatory to translate these molecular data into more effective treatments and better clinical management of PWS.

## Data availability statement

The datasets used and/or analyzed in the present study are available in the **Supplementary Material**. Raw data related to this study will be uploaded on Zenodo.org and available upon reasonable request to the corresponding author.

## Ethics statement

The studies involving humans were approved by Ethics Committee of Istituto Auxologico Italiano (Milan, Italy). The studies were conducted in accordance with the local legislation and institutional requirements. The participants provided their written informed consent to participate in this study.

## Author contributions

AR: Conceptualization, Writing – original draft, Writing – review & editing. EP: Data curation, Formal analysis, Methodology, Writing – review & editing. CF: Formal analysis, Writing – review & editing. DC: Data curation, Writing – review & editing. AB: Data curation, Writing – review & editing. GG: Investigation, Writing – review & editing. SM: Data curation, Writing – review & editing. SC: Writing – review & editing. SF: Funding acquisition, Methodology, Writing – review & editing. AS: Conceptualization, Funding acquisition, Investigation, Writing – review & editing.
