# Supplementary material for: Metabolomic profiling of Prader-Willi syndrome compared with essential obesity
Source: Front Endocrinol (Lausanne). 2024 May 15;15:1386265. doi: 10.3389/fendo.2024.1386265 (PMC11133515; doi:10.3389/fendo.2024.1386265)
Supplement: Supplementary file 1 [file DataSheet_1.zip › Supplementary Material/Legend to figure S1.docx]

**Legend to figures**

Figure S1. Box-plots summarizing the distribution of the 28 metabolites (µM) with the lowest FDR p-value in the Tobit multivariable linear regression models (<0.05) by case-control status (i.e., PWS vs. EOB).
