# Supplementary material for: Metabolomic profiling of Prader-Willi syndrome compared with essential obesity
Source: Front Endocrinol (Lausanne). 2024 May 15;15:1386265. doi: 10.3389/fendo.2024.1386265 (PMC11133515; doi:10.3389/fendo.2024.1386265)
Supplement: Supplementary file 1 [file DataSheet_1.zip › Supplementary Material/Legend to tables S1-S2-S3.docx]

**Legend to tables**

Tab. S1. Descriptive statistics of plasma metabolites’ concentrations in PWS and EOB groups.

Please, note that concentrations < LOD were replaced by the corresponding LOD/2 values.

LOD = Limit Of Detection

LB = Lower Bound = LOD/2

Tab. S2. Models of univariate Tobit linear regression, used to evaluate the association of Prader-Willi syndrome (PWS) with metabolites expressed in at least 20% of the population.

Tab. S3. Multivariable Tobit regression models used to evaluate the association of PWS with metabolites expressed in at least 20% of the population. Differently from Tab. 2, included in the article, all differences, i.e., the statistically significant and non significant metabolites, are here-in reported.
