# Supplementary material for: Metabolomic profiling of Prader-Willi syndrome compared with essential obesity
Source: Front Endocrinol (Lausanne). 2024 May 15;15:1386265. doi: 10.3389/fendo.2024.1386265 (PMC11133515; doi:10.3389/fendo.2024.1386265)
Supplement: Supplementary file 1 [file DataSheet_1.zip › Supplementary Material/Tab. S3.docx]

Tab. S3.

| **Metabolite** | **N obs < LB** | **PWS (N=32)** | | | **EOB (N=32)** | | | **FC** | **P-value** | **FDR P-value** |
| --- | --- | --- | --- | --- | --- | --- | --- | --- | --- | --- |
|  |  | Geometric Mean | 95% CI | | Geometric Mean | 95% CI | |  |  |  |
| C10 | 9 | 1,983 | 1,371 | 2,869 | 0,579 | 0,402 | 0,832 | 3,43 | <0.0001 | 0,0001 |
| lysoPC a C20:3 | 0 | 1,722 | 1,243 | 2,386 | 0,608 | 0,444 | 0,831 | 2,83 | <0.0001 | 0,0001 |
| C14:1 | 20 | 1,808 | 1,141 | 2,866 | 0,405 | 0,249 | 0,658 | 4,47 | <0.0001 | 0,0002 |
| lysoPC a C17:0 | 9 | 1,945 | 1,326 | 2,854 | 0,604 | 0,415 | 0,879 | 3,22 | <0.0001 | 0,0002 |
| C5 | 17 | 1,563 | 1,010 | 2,419 | 0,407 | 0,260 | 0,639 | 3,84 | <0.0001 | 0,0003 |
| lysoPC a C18:2 | 0 | 1,512 | 1,073 | 2,132 | 0,607 | 0,437 | 0,845 | 2,49 | 0,0001 | 0,0016 |
| lysoPC a C16:1 | 0 | 1,532 | 1,080 | 2,173 | 0,613 | 0,438 | 0,857 | 2,50 | 0,0001 | 0,0017 |
| lysoPC_s | 0 | 1,544 | 1,082 | 2,203 | 0,670 | 0,476 | 0,942 | 2,31 | 0,0004 | 0,0079 |
| PC ae C36:4 | 0 | 1,582 | 1,143 | 2,190 | 0,756 | 0,553 | 1,032 | 2,09 | 0,0006 | 0,0108 |
| lysoPC a C16:0 | 0 | 1,564 | 1,092 | 2,239 | 0,703 | 0,498 | 0,993 | 2,22 | 0,0008 | 0,0127 |
| PC ae C38:5 | 0 | 1,615 | 1,151 | 2,267 | 0,766 | 0,553 | 1,061 | 2,11 | 0,0009 | 0,0133 |
| PC ae C32:1 | 0 | 1,499 | 1,081 | 2,079 | 0,739 | 0,540 | 1,011 | 2,03 | 0,0011 | 0,0145 |
| lysoPC a C18:1 | 0 | 1,385 | 0,969 | 1,980 | 0,642 | 0,456 | 0,905 | 2,16 | 0,0011 | 0,0145 |
| PC aa C34:1 | 0 | 1,550 | 1,125 | 2,135 | 0,786 | 0,578 | 1,070 | 1,97 | 0,0014 | 0,0163 |
| PC aa C36:3 | 0 | 1,530 | 1,105 | 2,119 | 0,776 | 0,568 | 1,061 | 1,97 | 0,0016 | 0,0177 |
| PC ae C36:3 | 0 | 1,566 | 1,121 | 2,189 | 0,794 | 0,576 | 1,095 | 1,97 | 0,0021 | 0,0216 |
| PC aa C36:2 | 0 | 1,647 | 1,163 | 2,332 | 0,815 | 0,584 | 1,139 | 2,02 | 0,0022 | 0,0216 |
| lysoPC a C26:0 | 27 | 1,163 | 0,631 | 2,144 | 0,320 | 0,164 | 0,623 | 3,64 | 0,0023 | 0,0216 |
| PC aa_s | 0 | 1,523 | 1,099 | 2,112 | 0,797 | 0,582 | 1,090 | 1,91 | 0,0027 | 0,0236 |
| SM (OH) C22:2 | 23 | 1,401 | 0,799 | 2,458 | 0,463 | 0,259 | 0,830 | 3,02 | 0,0044 | 0,0329 |
| PC ae C36:5 | 0 | 1,501 | 1,067 | 2,111 | 0,789 | 0,569 | 1,095 | 1,90 | 0,0044 | 0,0329 |
| PC ae C42:0 | 2 | 1,287 | 0,906 | 1,830 | 0,666 | 0,474 | 0,934 | 1,93 | 0,0046 | 0,0329 |
| PC ae C34:1 | 0 | 1,488 | 1,075 | 2,062 | 0,811 | 0,593 | 1,110 | 1,83 | 0,0049 | 0,0329 |
| C3-DC (C4-OH) | 30 | 1,112 | 0,576 | 2,148 | 0,292 | 0,137 | 0,622 | 3,81 | 0,0049 | 0,0329 |
| PC ae C36:0 | 0 | 1,442 | 1,013 | 2,054 | 0,748 | 0,533 | 1,050 | 1,93 | 0,0049 | 0,0329 |
| PC aa C32:0 | 0 | 1,484 | 1,082 | 2,037 | 0,834 | 0,615 | 1,130 | 1,78 | 0,0058 | 0,0372 |
| PC ae C34:2 | 0 | 1,503 | 1,074 | 2,103 | 0,821 | 0,595 | 1,134 | 1,83 | 0,0065 | 0,0400 |
| PC aa C38:5 | 0 | 1,528 | 1,077 | 2,167 | 0,821 | 0,587 | 1,148 | 1,86 | 0,0072 | 0,0426 |
| PC ae C32:2 | 0 | 1,366 | 0,958 | 1,948 | 0,768 | 0,546 | 1,079 | 1,78 | 0,0140 | 0,0803 |
| PC aa C40:1 | 4 | 1,318 | 0,907 | 1,913 | 0,725 | 0,506 | 1,038 | 1,82 | 0,0153 | 0,0806 |
| PC ae C38:6 | 0 | 1,450 | 1,016 | 2,070 | 0,820 | 0,583 | 1,155 | 1,77 | 0,0155 | 0,0806 |
| PC aa C36:4 | 0 | 1,451 | 1,030 | 2,042 | 0,839 | 0,604 | 1,166 | 1,73 | 0,0155 | 0,0806 |
| C16:1 | 45 | 0,313 | 0,088 | 1,107 | 0,032 | 0,005 | 0,230 | 9,71 | 0,0174 | 0,0839 |
| C18:1 | 0 | 1,229 | 0,852 | 1,773 | 0,692 | 0,487 | 0,984 | 1,78 | 0,0176 | 0,0839 |
| lysoPC a C20:4 | 0 | 1,293 | 0,893 | 1,874 | 0,723 | 0,507 | 1,032 | 1,79 | 0,0177 | 0,0839 |
| lysoPC a C28:0 | 51 | 0,167 | 0,024 | 1,155 | 0,005 | 0,000 | 0,135 | 36,78 | 0,0193 | 0,0892 |
| C8 | 0 | 1,442 | 1,000 | 2,079 | 0,824 | 0,580 | 1,171 | 1,75 | 0,0208 | 0,0932 |
| PC aa C32:3 | 0 | 1,235 | 0,871 | 1,750 | 0,728 | 0,521 | 1,018 | 1,70 | 0,0220 | 0,0961 |
| PC ae C36:2 | 0 | 1,475 | 1,064 | 2,046 | 0,917 | 0,670 | 1,256 | 1,61 | 0,0280 | 0,1173 |
| PC ae C44:5 | 0 | 1,332 | 0,927 | 1,915 | 0,791 | 0,558 | 1,121 | 1,68 | 0,0300 | 0,1173 |
| PC ae C44:6 | 0 | 1,376 | 0,947 | 2,001 | 0,806 | 0,562 | 1,154 | 1,71 | 0,0304 | 0,1173 |
| PC aa C38:1 | 0 | 1,302 | 0,916 | 1,851 | 0,788 | 0,562 | 1,105 | 1,65 | 0,0307 | 0,1173 |
| PC aa C34:2 | 0 | 1,351 | 0,964 | 1,895 | 0,835 | 0,603 | 1,155 | 1,62 | 0,0312 | 0,1173 |
| C18:2 | 7 | 1,241 | 0,818 | 1,884 | 0,680 | 0,452 | 1,023 | 1,82 | 0,0312 | 0,1173 |
| PC aa C34:3 | 0 | 1,311 | 0,917 | 1,873 | 0,790 | 0,560 | 1,113 | 1,66 | 0,0318 | 0,1173 |
| C14:2 | 39 | 0,704 | 0,273 | 1,816 | 0,179 | 0,060 | 0,532 | 3,93 | 0,0339 | 0,1223 |
| PC ae C34:3 | 0 | 1,363 | 0,963 | 1,929 | 0,844 | 0,604 | 1,177 | 1,62 | 0,0365 | 0,1291 |
| PC aa C40:5 | 0 | 1,233 | 0,845 | 1,800 | 0,739 | 0,514 | 1,062 | 1,67 | 0,0402 | 0,1372 |
| PC aa C38:0 | 0 | 1,247 | 0,872 | 1,782 | 0,769 | 0,545 | 1,083 | 1,62 | 0,0405 | 0,1372 |
| lysoPC a C18:0 | 0 | 1,277 | 0,876 | 1,862 | 0,769 | 0,535 | 1,104 | 1,66 | 0,0418 | 0,1389 |
| SM (OH) C24:1 | 5 | 1,419 | 0,952 | 2,114 | 0,831 | 0,566 | 1,220 | 1,71 | 0,0429 | 0,1395 |
| PC ae C38:4 | 0 | 1,395 | 0,985 | 1,975 | 0,891 | 0,638 | 1,244 | 1,57 | 0,0514 | 0,1640 |
| PC aa C38:3 | 0 | 1,282 | 0,895 | 1,837 | 0,809 | 0,572 | 1,142 | 1,59 | 0,0526 | 0,1644 |
| C6 (C4:1-DC) | 22 | 1,007 | 0,588 | 1,725 | 0,499 | 0,288 | 0,864 | 2,02 | 0,0535 | 0,1644 |
| PC aa C34:4 | 0 | 1,240 | 0,855 | 1,800 | 0,808 | 0,565 | 1,155 | 1,54 | 0,0816 | 0,2461 |
| PC aa C38:4 | 0 | 1,400 | 0,967 | 2,026 | 0,919 | 0,645 | 1,311 | 1,52 | 0,0853 | 0,2529 |
| lysoPC a C24:0 | 44 | 0,305 | 0,085 | 1,095 | 0,075 | 0,016 | 0,360 | 4,06 | 0,0904 | 0,2633 |
| Creatinine | 0 | 0,777 | 0,540 | 1,119 | 1,159 | 0,816 | 1,644 | 0,67 | 0,0974 | 0,2775 |
| PC aa C36:0 | 0 | 1,250 | 0,870 | 1,796 | 0,842 | 0,595 | 1,192 | 1,48 | 0,0986 | 0,2775 |
| PC ae_s | 0 | 1,273 | 0,900 | 1,800 | 0,883 | 0,633 | 1,231 | 1,44 | 0,1104 | 0,2955 |
| C16:2 | 0 | 0,769 | 0,537 | 1,100 | 1,122 | 0,795 | 1,582 | 0,69 | 0,1109 | 0,2955 |
| PC ae C40:1 | 1 | 1,280 | 0,887 | 1,848 | 0,870 | 0,612 | 1,238 | 1,47 | 0,1116 | 0,2955 |
| H1 | 0 | 1,289 | 0,903 | 1,839 | 0,889 | 0,632 | 1,251 | 1,45 | 0,1138 | 0,2955 |
| PC aa C36:5 | 0 | 1,238 | 0,856 | 1,791 | 0,842 | 0,590 | 1,200 | 1,47 | 0,1139 | 0,2955 |
| C16:1-OH | 0 | 0,768 | 0,536 | 1,101 | 1,114 | 0,789 | 1,574 | 0,69 | 0,1182 | 0,2994 |
| PC aa C32:2 | 0 | 1,191 | 0,820 | 1,730 | 0,811 | 0,566 | 1,160 | 1,47 | 0,1190 | 0,2994 |
| PC aa C32:1 | 0 | 1,242 | 0,862 | 1,790 | 0,866 | 0,610 | 1,230 | 1,43 | 0,1359 | 0,3366 |
| PC ae C34:0 | 0 | 1,347 | 0,954 | 1,902 | 0,963 | 0,692 | 1,341 | 1,40 | 0,1412 | 0,3448 |
| PC ae C38:0 | 0 | 1,262 | 0,874 | 1,823 | 0,894 | 0,629 | 1,273 | 1,41 | 0,1561 | 0,3755 |
| SM C16:1 | 0 | 1,178 | 0,821 | 1,689 | 0,842 | 0,595 | 1,190 | 1,40 | 0,1587 | 0,3763 |
| SM (OH) C22:1 | 11 | 1,179 | 0,743 | 1,871 | 0,770 | 0,491 | 1,207 | 1,53 | 0,1661 | 0,3884 |
| PC aa C38:6 | 0 | 1,304 | 0,900 | 1,890 | 0,945 | 0,662 | 1,350 | 1,38 | 0,1893 | 0,4364 |
| PC ae C38:1 | 0 | 1,185 | 0,838 | 1,677 | 0,879 | 0,630 | 1,226 | 1,35 | 0,1924 | 0,4376 |
| PC aa C42:6 | 20 | 1,002 | 0,590 | 1,699 | 0,637 | 0,376 | 1,079 | 1,57 | 0,2017 | 0,4471 |
| C18 | 27 | 0,806 | 0,413 | 1,571 | 0,458 | 0,233 | 0,901 | 1,76 | 0,2020 | 0,4471 |
| Acylcarnitines_s | 0 | 1,210 | 0,823 | 1,779 | 0,888 | 0,613 | 1,285 | 1,36 | 0,2239 | 0,4713 |
| C5-OH (C3-DC-M) | 19 | 0,557 | 0,327 | 0,948 | 0,841 | 0,512 | 1,382 | 0,66 | 0,2243 | 0,4713 |
| Sphingolipids_s | 0 | 1,172 | 0,823 | 1,667 | 0,883 | 0,629 | 1,240 | 1,33 | 0,2263 | 0,4713 |
| PC aa C36:1 | 0 | 1,095 | 0,748 | 1,602 | 0,807 | 0,560 | 1,164 | 1,36 | 0,2271 | 0,4713 |
| PC aa C42:5 | 0 | 1,245 | 0,863 | 1,794 | 0,929 | 0,654 | 1,320 | 1,34 | 0,2271 | 0,4713 |
| PC aa C36:6 | 0 | 1,209 | 0,831 | 1,758 | 0,899 | 0,627 | 1,288 | 1,35 | 0,2315 | 0,4722 |
| PC ae C42:5 | 0 | 1,211 | 0,840 | 1,746 | 0,909 | 0,640 | 1,292 | 1,33 | 0,2355 | 0,4722 |
| Kynurenine | 1 | 0,905 | 0,620 | 1,321 | 1,217 | 0,847 | 1,750 | 0,74 | 0,2361 | 0,4722 |
| SM C18:1 | 0 | 1,155 | 0,799 | 1,670 | 0,867 | 0,608 | 1,235 | 1,33 | 0,2389 | 0,4722 |
| His | 0 | 0,797 | 0,549 | 1,156 | 1,063 | 0,743 | 1,520 | 0,75 | 0,2424 | 0,4734 |
| C0 | 0 | 1,193 | 0,812 | 1,753 | 0,890 | 0,615 | 1,287 | 1,34 | 0,2482 | 0,4792 |
| PC ae C42:4 | 0 | 1,157 | 0,803 | 1,666 | 0,886 | 0,624 | 1,258 | 1,31 | 0,2686 | 0,5076 |
| SM C18:0 | 0 | 1,179 | 0,821 | 1,693 | 0,905 | 0,640 | 1,282 | 1,30 | 0,2691 | 0,5076 |
| Spermine | 33 | 0,310 | 0,129 | 0,744 | 0,546 | 0,249 | 1,194 | 0,57 | 0,2782 | 0,5188 |
| Phe | 0 | 0,875 | 0,605 | 1,266 | 1,136 | 0,797 | 1,620 | 0,77 | 0,2851 | 0,5259 |
| Ac-Orn | 14 | 0,851 | 0,529 | 1,368 | 0,611 | 0,381 | 0,979 | 1,39 | 0,2939 | 0,5310 |
| PC ae C40:4 | 0 | 1,160 | 0,805 | 1,670 | 0,904 | 0,637 | 1,283 | 1,28 | 0,3020 | 0,5310 |
| PC aa C42:1 | 0 | 1,173 | 0,810 | 1,699 | 0,912 | 0,639 | 1,301 | 1,29 | 0,3031 | 0,5310 |
| Trp | 0 | 0,829 | 0,573 | 1,200 | 1,067 | 0,748 | 1,521 | 0,78 | 0,3036 | 0,5310 |
| PC aa C40:6 | 0 | 1,188 | 0,812 | 1,738 | 0,919 | 0,638 | 1,324 | 1,29 | 0,3070 | 0,5310 |
| SM C20:2 | 0 | 1,187 | 0,838 | 1,683 | 0,938 | 0,671 | 1,312 | 1,27 | 0,3071 | 0,5310 |
| Biogenic Amines_s | 0 | 0,788 | 0,537 | 1,157 | 1,017 | 0,704 | 1,470 | 0,77 | 0,3141 | 0,5375 |
| PC ae C40:6 | 0 | 1,222 | 0,844 | 1,768 | 0,962 | 0,675 | 1,373 | 1,27 | 0,3294 | 0,5580 |
| SM C24:0 | 0 | 0,898 | 0,610 | 1,322 | 1,148 | 0,792 | 1,664 | 0,78 | 0,3370 | 0,5651 |
| C16-OH | 43 | 0,099 | 0,024 | 0,412 | 0,189 | 0,056 | 0,635 | 0,52 | 0,3419 | 0,5652 |
| PC ae C40:2 | 0 | 1,141 | 0,794 | 1,639 | 0,911 | 0,643 | 1,290 | 1,25 | 0,3470 | 0,5652 |
| Putrescine | 1 | 1,133 | 0,789 | 1,629 | 0,905 | 0,639 | 1,283 | 1,25 | 0,3487 | 0,5652 |
| Leu | 0 | 0,909 | 0,629 | 1,314 | 1,139 | 0,799 | 1,622 | 0,80 | 0,3561 | 0,5652 |
| ADMA | 33 | 0,685 | 0,315 | 1,491 | 0,431 | 0,198 | 0,938 | 1,59 | 0,3564 | 0,5652 |
| C3 | 0 | 0,862 | 0,585 | 1,271 | 1,092 | 0,752 | 1,586 | 0,79 | 0,3575 | 0,5652 |
| SM C16:0 | 0 | 1,128 | 0,783 | 1,624 | 0,906 | 0,638 | 1,286 | 1,24 | 0,3642 | 0,5700 |
| SM C26:0 | 24 | 0,721 | 0,402 | 1,294 | 0,512 | 0,288 | 0,912 | 1,41 | 0,3674 | 0,5700 |
| C5-DC (C6-OH) | 20 | 0,828 | 0,474 | 1,446 | 0,597 | 0,346 | 1,031 | 1,39 | 0,3729 | 0,5732 |
| PC ae C44:4 | 0 | 1,135 | 0,797 | 1,615 | 0,929 | 0,662 | 1,303 | 1,22 | 0,3904 | 0,5945 |
| Spermidine | 3 | 0,761 | 0,511 | 1,133 | 0,952 | 0,649 | 1,394 | 0,80 | 0,3956 | 0,5970 |
| total DMA | 47 | 0,075 | 0,012 | 0,481 | 0,166 | 0,034 | 0,825 | 0,45 | 0,4000 | 0,5983 |
| C4:1 | 39 | 0,223 | 0,077 | 0,644 | 0,362 | 0,141 | 0,926 | 0,62 | 0,4164 | 0,6117 |
| Ile | 0 | 0,918 | 0,636 | 1,324 | 1,116 | 0,785 | 1,586 | 0,82 | 0,4199 | 0,6117 |
| Met-SO | 8 | 0,845 | 0,546 | 1,307 | 1,064 | 0,703 | 1,611 | 0,79 | 0,4201 | 0,6117 |
| PC ae C40:3 | 0 | 1,124 | 0,778 | 1,622 | 0,929 | 0,653 | 1,321 | 1,21 | 0,4326 | 0,6245 |
| C18:1-OH | 50 | 0,027 | 0,002 | 0,343 | 0,065 | 0,008 | 0,540 | 0,41 | 0,4395 | 0,6289 |
| lysoPC a C28:1 | 7 | 0,976 | 0,632 | 1,506 | 0,785 | 0,516 | 1,193 | 1,24 | 0,4480 | 0,6356 |
| Thr | 0 | 1,029 | 0,703 | 1,506 | 0,853 | 0,591 | 1,230 | 1,21 | 0,4562 | 0,6402 |
| PC aa C40:4 | 0 | 1,111 | 0,767 | 1,611 | 0,927 | 0,649 | 1,324 | 1,20 | 0,4600 | 0,6402 |
| PC ae C30:0 | 29 | 0,802 | 0,401 | 1,606 | 0,573 | 0,284 | 1,159 | 1,40 | 0,4661 | 0,6402 |
| Histamine | 14 | 1,116 | 0,688 | 1,809 | 0,884 | 0,554 | 1,409 | 1,26 | 0,4667 | 0,6402 |
| Arg | 0 | 0,961 | 0,666 | 1,386 | 1,139 | 0,802 | 1,619 | 0,84 | 0,4825 | 0,6485 |
| Glu | 0 | 0,930 | 0,647 | 1,336 | 1,100 | 0,776 | 1,558 | 0,85 | 0,4837 | 0,6485 |
| PC ae C42:2 | 0 | 1,128 | 0,782 | 1,626 | 0,952 | 0,670 | 1,353 | 1,18 | 0,4844 | 0,6485 |
| PC aa C42:0 | 0 | 1,114 | 0,769 | 1,613 | 0,947 | 0,664 | 1,352 | 1,18 | 0,5083 | 0,6750 |
| C4 | 21 | 0,604 | 0,344 | 1,059 | 0,765 | 0,445 | 1,317 | 0,79 | 0,5124 | 0,6751 |
| Orn | 0 | 0,971 | 0,661 | 1,428 | 0,831 | 0,574 | 1,203 | 1,17 | 0,5406 | 0,6999 |
| SM C22:3 | 33 | 0,763 | 0,354 | 1,647 | 0,560 | 0,261 | 1,204 | 1,36 | 0,5419 | 0,6999 |
| SM C24:1 | 0 | 1,096 | 0,748 | 1,606 | 0,940 | 0,652 | 1,357 | 1,17 | 0,5439 | 0,6999 |
| Cit | 0 | 0,971 | 0,661 | 1,426 | 0,834 | 0,576 | 1,207 | 1,16 | 0,5510 | 0,7035 |
| PC aa C30:0 | 0 | 1,040 | 0,717 | 1,509 | 0,903 | 0,632 | 1,291 | 1,15 | 0,5659 | 0,7171 |
| PC ae C40:5 | 0 | 1,112 | 0,772 | 1,602 | 0,979 | 0,689 | 1,390 | 1,14 | 0,5976 | 0,7516 |
| PC ae C44:3 | 0 | 1,111 | 0,773 | 1,595 | 0,982 | 0,693 | 1,390 | 1,13 | 0,6062 | 0,7567 |
| SM (OH) C16:1 | 0 | 1,107 | 0,776 | 1,580 | 0,984 | 0,699 | 1,384 | 1,13 | 0,6151 | 0,7590 |
| Aminoacids_s | 0 | 0,925 | 0,635 | 1,348 | 1,047 | 0,730 | 1,504 | 0,88 | 0,6173 | 0,7590 |
| PC ae C42:1 | 0 | 1,100 | 0,760 | 1,591 | 0,984 | 0,690 | 1,403 | 1,12 | 0,6488 | 0,7784 |
| Taurine | 0 | 0,839 | 0,571 | 1,232 | 0,941 | 0,650 | 1,362 | 0,89 | 0,6506 | 0,7784 |
| PC ae C38:3 | 0 | 1,112 | 0,773 | 1,600 | 0,998 | 0,704 | 1,415 | 1,11 | 0,6530 | 0,7784 |
| PC aa C28:1 | 0 | 0,892 | 0,608 | 1,309 | 1,000 | 0,691 | 1,445 | 0,89 | 0,6535 | 0,7784 |
| C3:1 | 40 | 0,210 | 0,070 | 0,635 | 0,276 | 0,102 | 0,747 | 0,76 | 0,6567 | 0,7784 |
| SM (OH) C14:1 | 0 | 1,041 | 0,726 | 1,493 | 0,938 | 0,663 | 1,326 | 1,11 | 0,6612 | 0,7784 |
| PC aa C40:3 | 0 | 1,100 | 0,759 | 1,593 | 0,993 | 0,696 | 1,417 | 1,11 | 0,6761 | 0,7849 |
| PC ae C38:2 | 0 | 1,085 | 0,752 | 1,565 | 0,980 | 0,689 | 1,394 | 1,11 | 0,6761 | 0,7849 |
| Lys | 0 | 0,967 | 0,659 | 1,417 | 1,072 | 0,742 | 1,548 | 0,90 | 0,6825 | 0,7868 |
| Val | 0 | 0,944 | 0,648 | 1,377 | 1,043 | 0,726 | 1,498 | 0,91 | 0,6921 | 0,7876 |
| Serotonin | 6 | 0,865 | 0,562 | 1,330 | 0,967 | 0,642 | 1,458 | 0,89 | 0,6927 | 0,7876 |
| Ser | 0 | 0,916 | 0,626 | 1,342 | 1,011 | 0,701 | 1,458 | 0,91 | 0,6976 | 0,7877 |
| Gln | 0 | 0,967 | 0,655 | 1,428 | 0,896 | 0,616 | 1,303 | 1,08 | 0,7680 | 0,8614 |
| SM C26:1 | 22 | 0,827 | 0,457 | 1,496 | 0,745 | 0,421 | 1,320 | 1,11 | 0,7878 | 0,8776 |
| Pro | 0 | 1,046 | 0,712 | 1,536 | 1,117 | 0,772 | 1,615 | 0,94 | 0,7963 | 0,8813 |
| Gly | 0 | 1,005 | 0,685 | 1,475 | 0,945 | 0,654 | 1,366 | 1,06 | 0,8084 | 0,8831 |
| PC aa C42:4 | 0 | 0,980 | 0,672 | 1,428 | 1,041 | 0,725 | 1,494 | 0,94 | 0,8086 | 0,8831 |
| PC aa C42:2 | 0 | 1,061 | 0,723 | 1,559 | 1,002 | 0,693 | 1,450 | 1,06 | 0,8223 | 0,8922 |
| Tyr | 0 | 0,974 | 0,672 | 1,414 | 1,026 | 0,718 | 1,467 | 0,95 | 0,8330 | 0,8946 |
| Ala | 0 | 0,957 | 0,652 | 1,405 | 1,009 | 0,698 | 1,459 | 0,95 | 0,8353 | 0,8946 |
| PC ae C30:2 | 0 | 0,962 | 0,651 | 1,422 | 1,008 | 0,693 | 1,468 | 0,95 | 0,8547 | 0,9095 |
| C2 | 0 | 1,007 | 0,683 | 1,485 | 1,051 | 0,724 | 1,527 | 0,96 | 0,8673 | 0,9171 |
| C16 | 15 | 0,763 | 0,458 | 1,270 | 0,804 | 0,494 | 1,308 | 0,95 | 0,8743 | 0,9186 |
| PC aa C40:2 | 0 | 1,052 | 0,726 | 1,524 | 1,015 | 0,711 | 1,450 | 1,04 | 0,8864 | 0,9235 |
| PC ae C42:3 | 0 | 1,021 | 0,707 | 1,474 | 0,987 | 0,694 | 1,405 | 1,03 | 0,8901 | 0,9235 |
| Met | 0 | 0,933 | 0,638 | 1,365 | 0,907 | 0,629 | 1,307 | 1,03 | 0,9098 | 0,9381 |
| PC ae C36:1 | 0 | 1,062 | 0,737 | 1,531 | 1,040 | 0,732 | 1,478 | 1,02 | 0,9311 | 0,9541 |
| PC ae C30:1 | 50 | 0,052 | 0,005 | 0,531 | 0,056 | 0,006 | 0,526 | 0,92 | 0,9426 | 0,9566 |
| Asp | 0 | 1,013 | 0,687 | 1,493 | 1,031 | 0,710 | 1,497 | 0,98 | 0,9451 | 0,9566 |
| PC aa C24:0 | 30 | 0,505 | 0,233 | 1,097 | 0,511 | 0,247 | 1,057 | 0,99 | 0,9808 | 0,9868 |
| Asn | 0 | 0,900 | 0,614 | 1,319 | 0,900 | 0,623 | 1,300 | 1,00 | 0,9988 | 0,9988 |

Sex, CRP and DBP were selected as adjustment covariates. LB=Lower Bound; FC=Fold-Change.
