# Supplementary figures and images for: Metabolomic profiling of Prader-Willi syndrome compared with essential obesity
Source: Front Endocrinol (Lausanne). 2024 May 15;15:1386265. doi: 10.3389/fendo.2024.1386265 (PMC11133515; doi:10.3389/fendo.2024.1386265)

## Slide 1
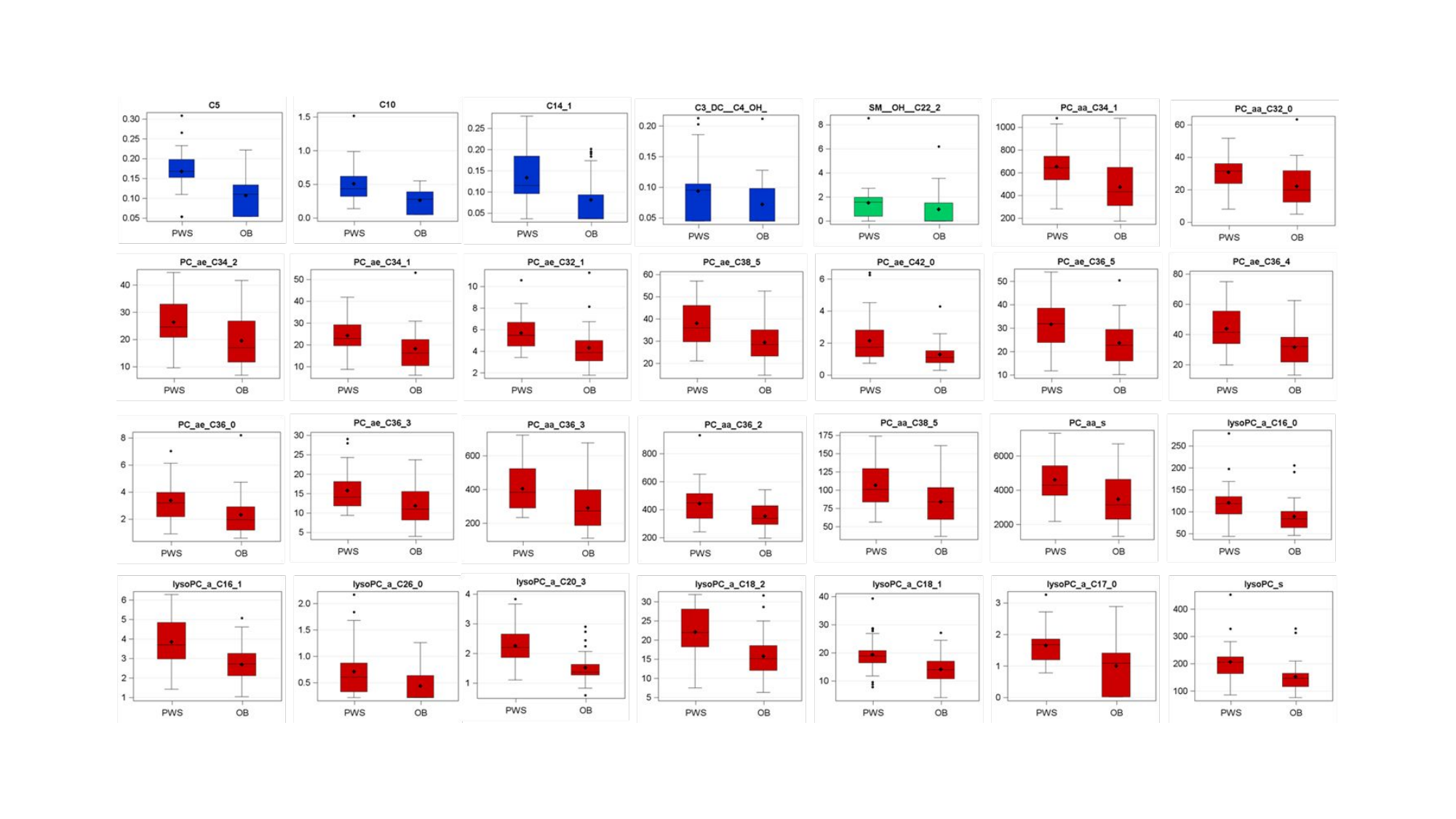

Supplement: Supplementary file 1 [file DataSheet_1.zip › Supplementary Material/Fig. S1.pptx]
